# 379. Potential clinical and economic impact of cutbacks in the United States President's Emergency Plan for AIDS Relief Program in South Africa: a modelling analysis

**DOI:** 10.1093/ofid/ofae631.010

**Published:** 2025-01-29

**Authors:** Aditya Gandhi, Linda-Gail Bekker, A David Paltiel, Emily P Hyle, Andrea L Ciaranello, Yogan Pillay, Kenneth A Freedberg, Anne M Neilan

**Affiliations:** NYU Grossman School of Medicine, New York, NY; Yale School of Public Health, New Haven, Connecticut; Massachusetts General Hospital, Boston, Massachusetts; Massachusetts General Hospital, Boston, Massachusetts; Stellenbosch University, Stellenbosch, Western Cape, South Africa; Massachusetts General Hospital, Boston, Massachusetts; Massachusetts General Hospital, Boston, Massachusetts

## Abstract

**Background:**

The US PEPFAR program is one of the most successful global health initiatives ever undertaken. However, congressional reauthorization is unclear. We evaluate the clinical and economic impact of scaling back PEPFAR funding ($460 million) from South Africa’s HIV budget ($2.60 billion) in 2024.

**Figure:**
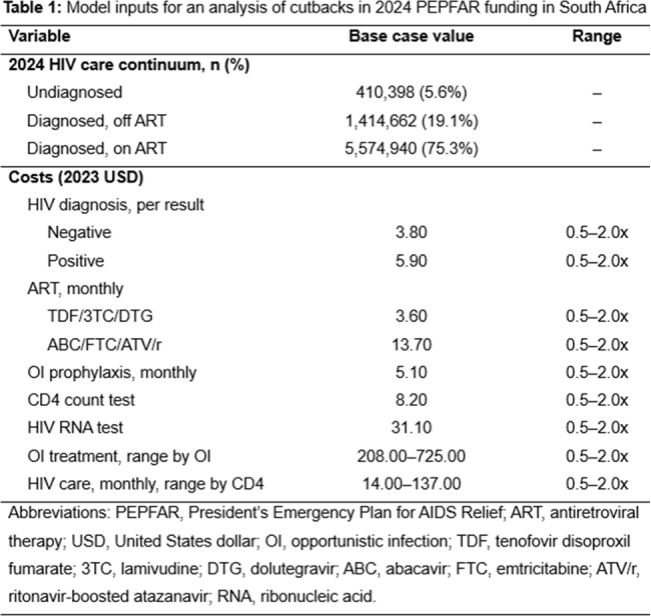

**Methods:**

Using the Cost-Effectiveness of Preventing AIDS Complications (CEPAC) microsimulation model, we examine 100% (PEPFAR_100%), 50% (PEPFAR_50%), and 0% (PEPFAR_0%) PEPFAR funding cutbacks among South African adults (HIV prevalence: 16.2%; incidence: 0.32/100PY [person-years]) using published HIV care continuum data (Table 1) and PEPFAR funding estimates. We model proportional decreases in HIV diagnosis (26.0, 24.3, 22.6/100PY), treatment (one-year engagement among people with HIV [PWH] on/initiating antiretroviral therapy: 92.2%/80.4%, 87.1%/76.0%, 82.0%/71.5%), and primary prevention (4.0%, 2.2%, 0.5% reduction in incidence with no programming [1.24/100PY]) (Table 2). We project new HIV infections and HIV-related deaths over 10 years, life expectancy and HIV-related lifetime costs in 2023 US dollars from a modified societal perspective. We vary key parameters and assumptions in sensitivity analyses.

**Figure:**
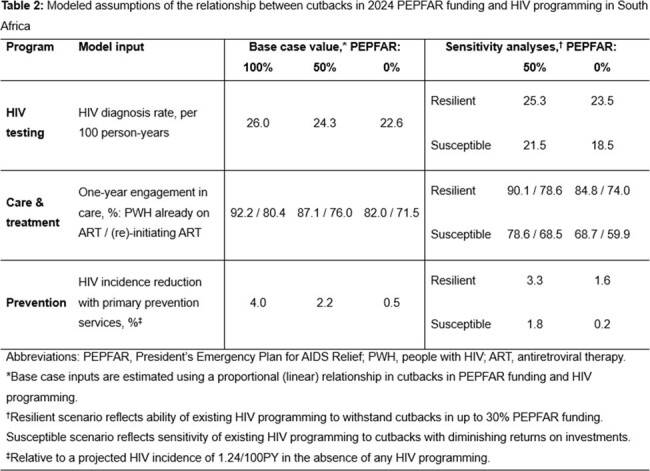

**Results:**

With current HIV programming (PEPFAR_100%), a projected 1,190,000 infections would occur over 10 years (Table 3); life expectancy would be 61.42 years for PWH with lifetime costs of $11,180/PWH. PEPFAR_50% and PEPFAR_0% would add 286,000 and 565,000 infections and worsen HIV care continuum outcomes (Figure 1). PWH would lose 2.02 and 3.71 life-years with nominal lifetime cost reductions of $620/PWH and $1,140/PWH that would be offset at the population level by more PWH requiring treatment for infection. In sensitivity analyses, clinical and epidemiologic impacts of scaling back PEPFAR would remain substantial.

**Figure:**
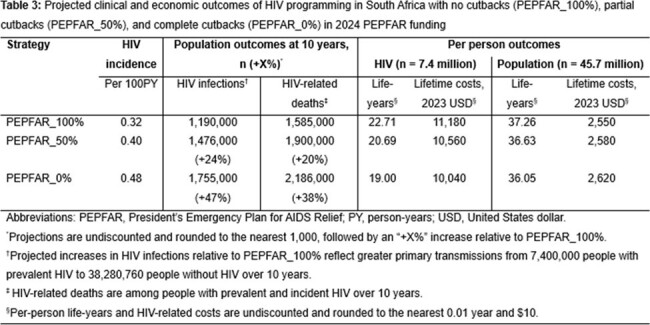

**Conclusion:**

Abruptly scaling back PEPFAR funding would have a striking, detrimental impact on the progress South Africa has made towards HIV epidemic control. Any total cost reductions would be short-lived and at the expense of up to an additional 565,000 new HIV infections and 601,000 HIV-related deaths in South Africa by 2034.

**Figure:**
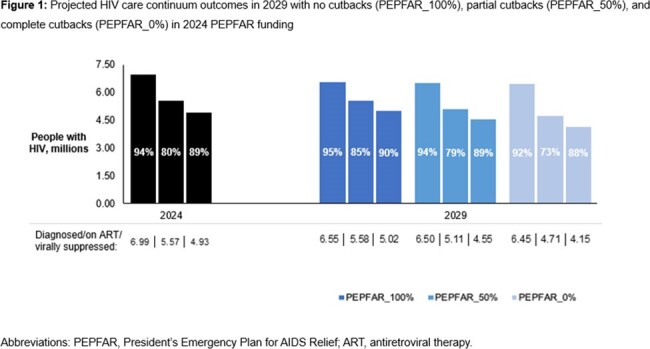

The estimated 2024 HIV care cascade (black bars) is based on published data. The projected HIV care cascade in 2029 is shown for PEPFAR_100% (dark blue bars), PEPFAR_50% (blue bars), and PEPFAR_0% (light blue bars). Each set of bars represents PWH who are aware of their HIV status, receiving ART, and virally suppressed (HIV RNA <20 copies/mL), respectively. The percentages shown are in respect to the conditional UNAIDS’ 95-95-95 epidemic targets (i.e., proportion of all PWH who are aware of their status, proportion of PWH aware of their status who are receiving ART, and proportion of PWH receiving ART who are virally suppressed). The estimated number of PWH alive in South Africa in 2024 is 7,400,000; the projected number of PWH alive in 2029 would be 6,913,000 with PEPFAR_100%, 6,950,000 with PEPFAR_50%, and 6,997,000 with PEPFAR_0%. The number of PWH associated with each bar is labeled at the bottom of Panel B and rounded to the nearest 10,000.

**Disclosures:**

**Linda-Gail Bekker**, Gilead Health Sciences: Honoraria|MSD (Pty) Ltd: Honoraria|ViiV Healthcare: Honoraria

